# Supplementary material for: A multi‐pore model of the blood–brain barrier tight junction strands recapitulates the permeability features of wild‐type and mutant claudin‐5
Source: Protein Sci. 2025 Aug 27;34(9):e70271. doi: 10.1002/pro.70271 (PMC12381782; doi:10.1002/pro.70271)
Supplement: Supplementary file 1 — Data S1. Supporting Information. [file PRO-34-e70271-s001.pdf]

Supplementary Information for:

A multi-pore model of the blood-brain barrier tight junction strands recapitulates the permeability features of wild-type and mutant claudin-5

Alessandro Berselli[1,2*§], Giulio Alberini[1,3*], Linda Cerioni[4], Fabio Benfenati[1,3°], Luca Maragliano[1,4°]

[1]Center for Synaptic Neuroscience and Technology (NSYN@UniGe), Istituto Italiano di Tecnologia, Largo Rosanna Benzi, 10, 16132, Genova, Italy

[2]Department of Experimental Medicine, Università degli Studi di Genova, Viale Benedetto XV, 3, 16132, Genova,

Italy

[3]IRCCS Ospedale Policlinico San Martino, Largo Rosanna Benzi, 10, 16132, Genova, Italy

[4]Department of Life and Environmental Sciences, Polytechnic University of Marche, Via Brecce Bianche, 60131, Ancona, Italy.

*Equal contribution

°Corresponding authors: fabio.benfenati@iit.it; l.maragliano@univpm.it

§Present address: Department of Chemical and Geological Sciences, University of Modena and Reggio Emilia (UNIMORE), Via Campi 103, 41125 Modena, Italy.

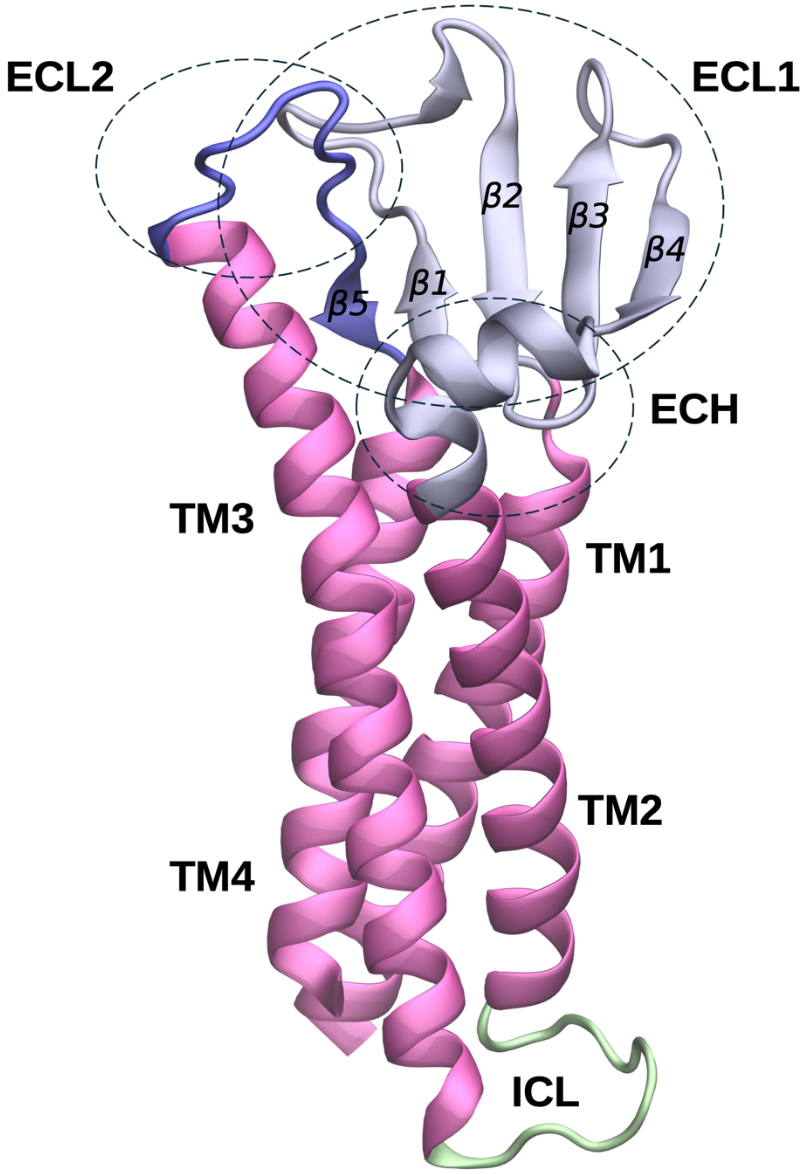

Figure S1. Three-dimensional structure of the Cldn5 monomer. *The three-dimensional structure of the Cldn5 monomer was reproduced with SWISS-MODEL (https://swissmodel.expasy.org) using the Cldn15 crystal as a template (PDB ID: 4P79). The structural domains are indicated with the nomenclature used in the main text and distinguished by their coloring: magenta for TM domains, silver for ECL1, blue for ECL2, and green for the intracellular loop (ICL).*

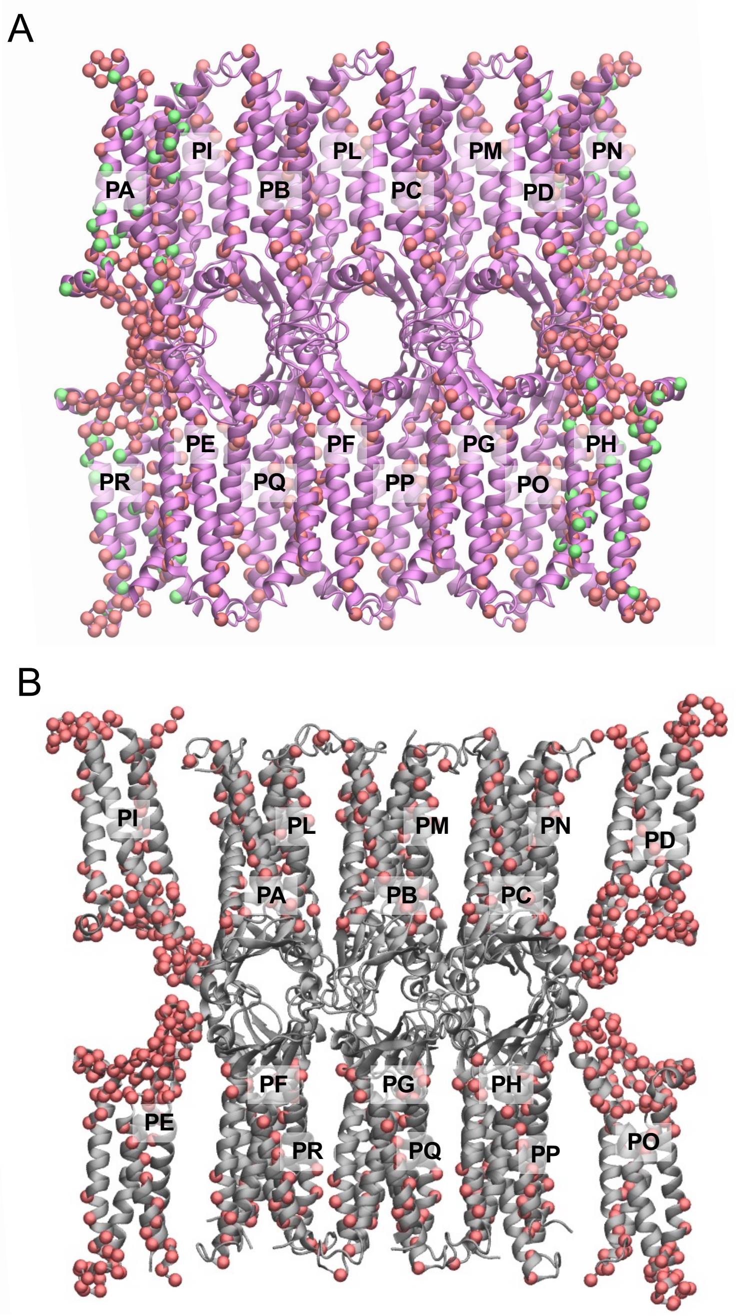

Figure S2. Positional restraints used in MD simulations of multi-Pore models. *Positionally restrained Cα atoms are shown as VdW spheres colored in red for the extended set and green for the restricted set. (A) multi-Pore I. (B) multi-Pore II.*

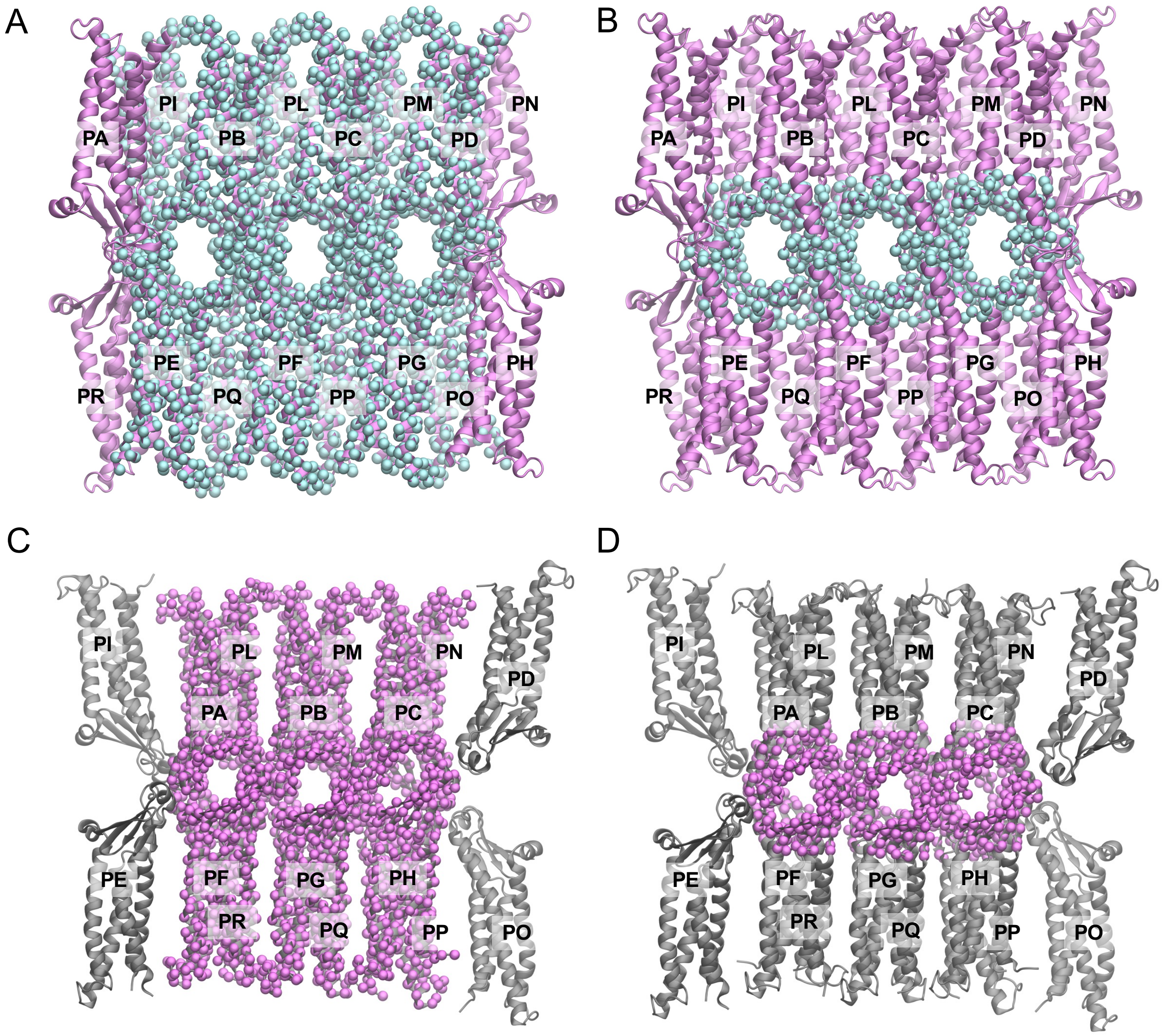

Figure S3. Atoms considered in the RMSD calculations. *VdW spheres are used to indicate the backbone atoms of the multi-Pore I (**A,** whole system, excluding peripheral protomers; **B,** paracellular domain) and multi-Pore II (**C,** whole system, excluding peripheral protomers; **D,** paracellular domain) models used for RMSD calculations.*

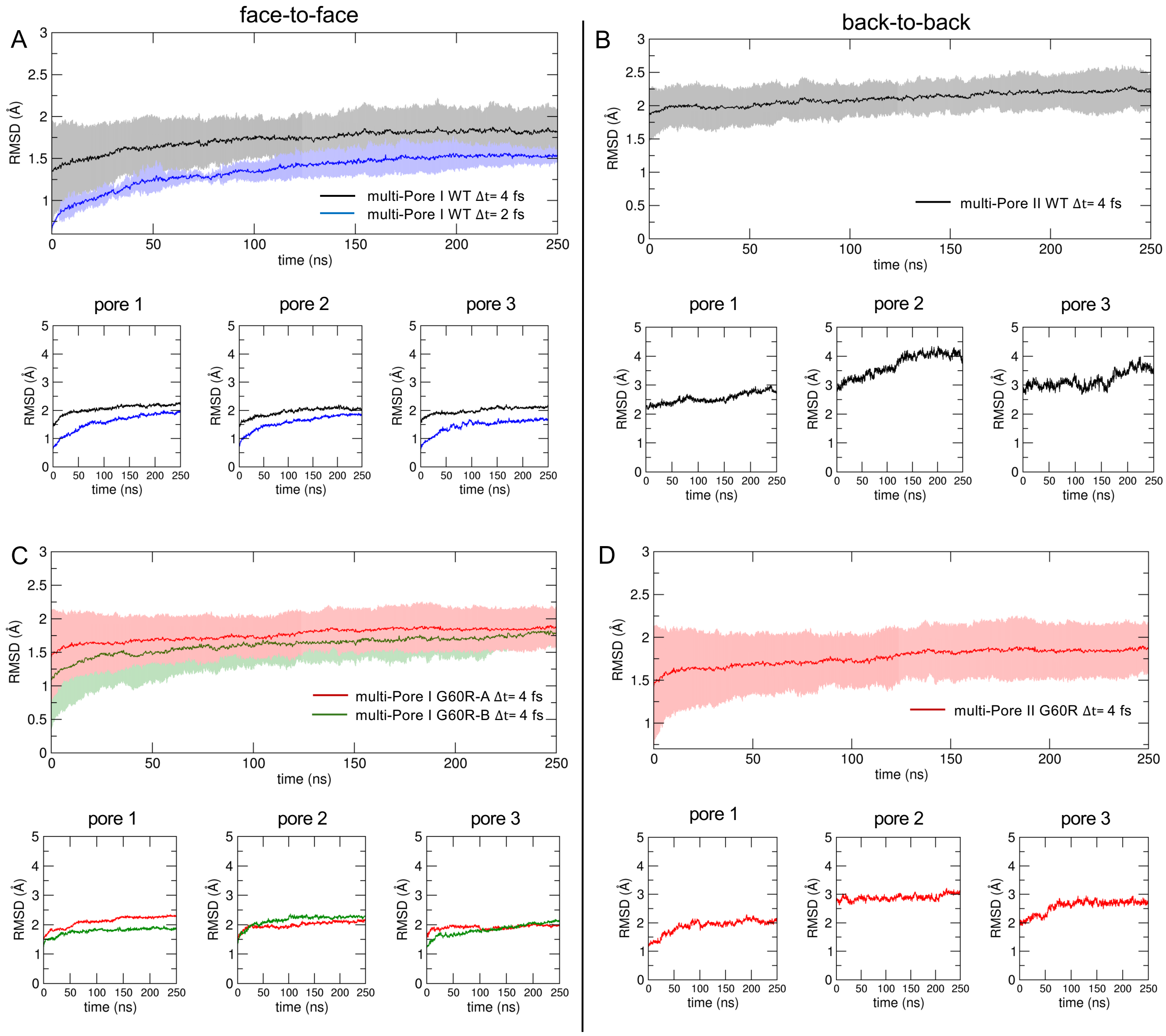

Figure S4. RMSD calculations of the benchmark replicas. A*, time evolution of backbone RMSD of multi-Pore I with HMR (black line) and standard masses (blue line) employing the extended set of restraints. RMSDs are calculated for the whole system (ECLs plus TM domains) and the three separate pores (ECLs only, lower panels).* ***B****, backbone RMSD of multi-Pore II with HMR (black line) and the extended set of restraints, calculated for the whole system and the three separate pores (lower panels).* ***C,*** *backbone RMSD of multi-Pore I mutated models with HMR and the extended set of restraints, whole system and individual pores (lower panels).* ***D,*** *backbone RMSD of mutated multi-Pore II with HMR and the extended set of restraints, whole system and individual pores (lower panels). In each panel, the average RMSD and, for the whole systems only, the standard deviation from three benchmark replicas are shown.*

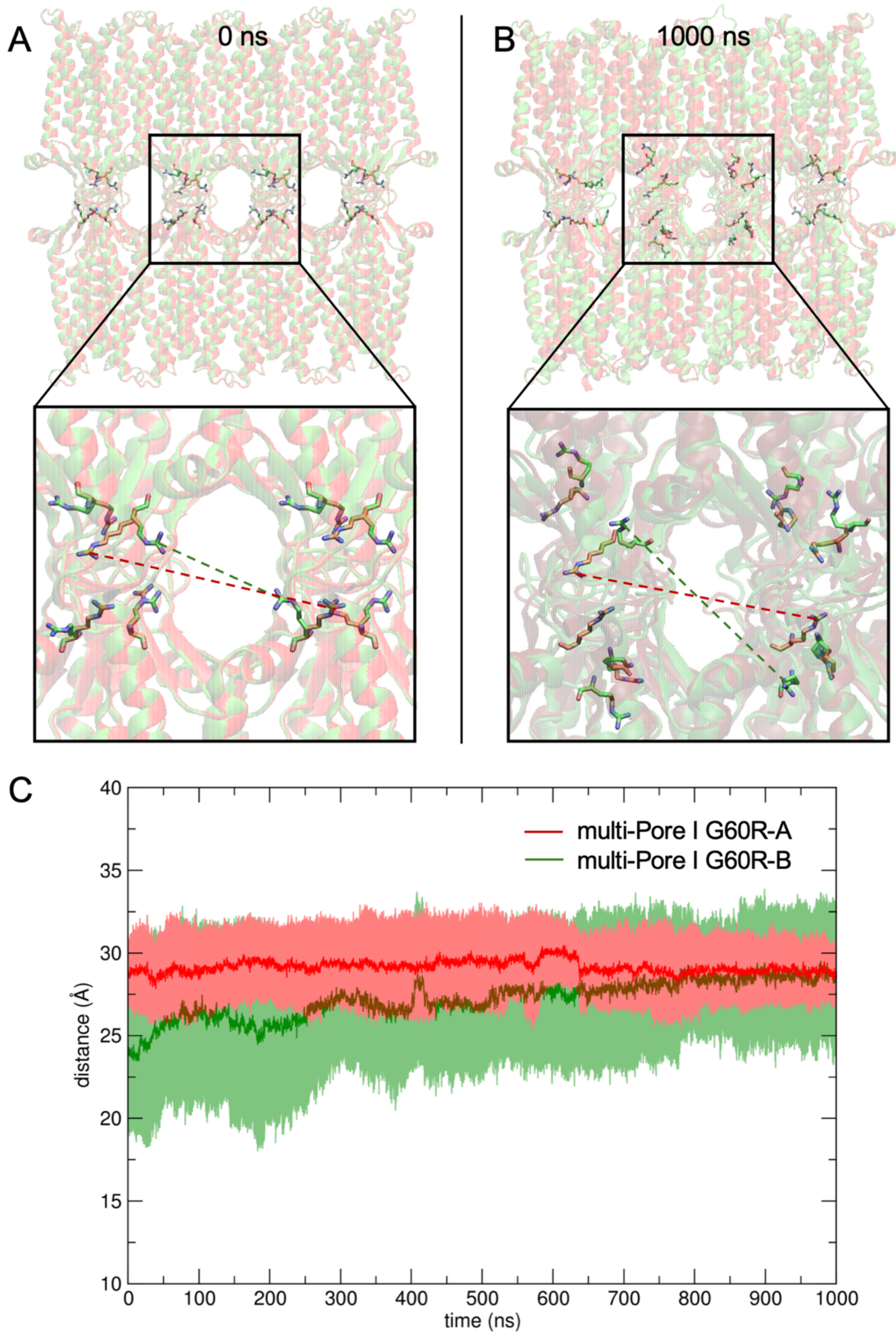

***Figure S5. Structural analysis of the G60R multi-Pore I models. A**, structures of the G60R-A and G60R-B multi-Pore-I like structures before MD simulations. For each panel, the R60 sidechains are shown in sticks. **B**, the same structures of the G60R-A and G60R-B systems at the end of the standard AA-MD simulations. **C**, average cross-distance between the R60 CZ-atom of multi-Pore I G60R-A (red trace) and G60R-B (green trace) during 1 µs-long MD simulations with HMR and the extended set of restraints. The average value and associated error are calculated as the mean and standard deviation using each pair of facing R60 residues across the three pores of each system.*

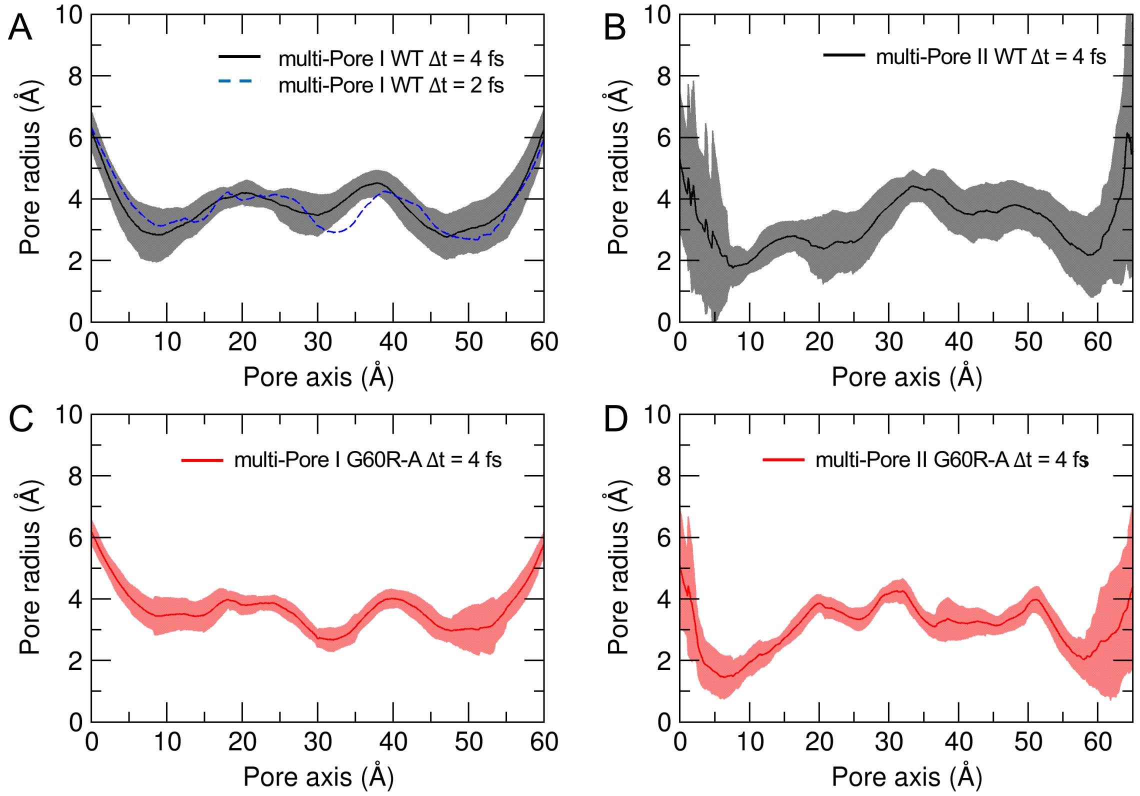

Figure S6. Pore radius profiles of the benchmark HMR replicas with the extended set of restraints. A*, WT multi-Pore I, calculated from simulations with HMR (black line) or standard atomic masses (blue dashed line).* ***B****, WT multi-Pore II (black line).* ***C****, G60R multi-Pore I (red line).* ***D****, G60R multi-Pore II (red line), compared with the single-Pore II from Ref.* 16 *(red dashed line).*

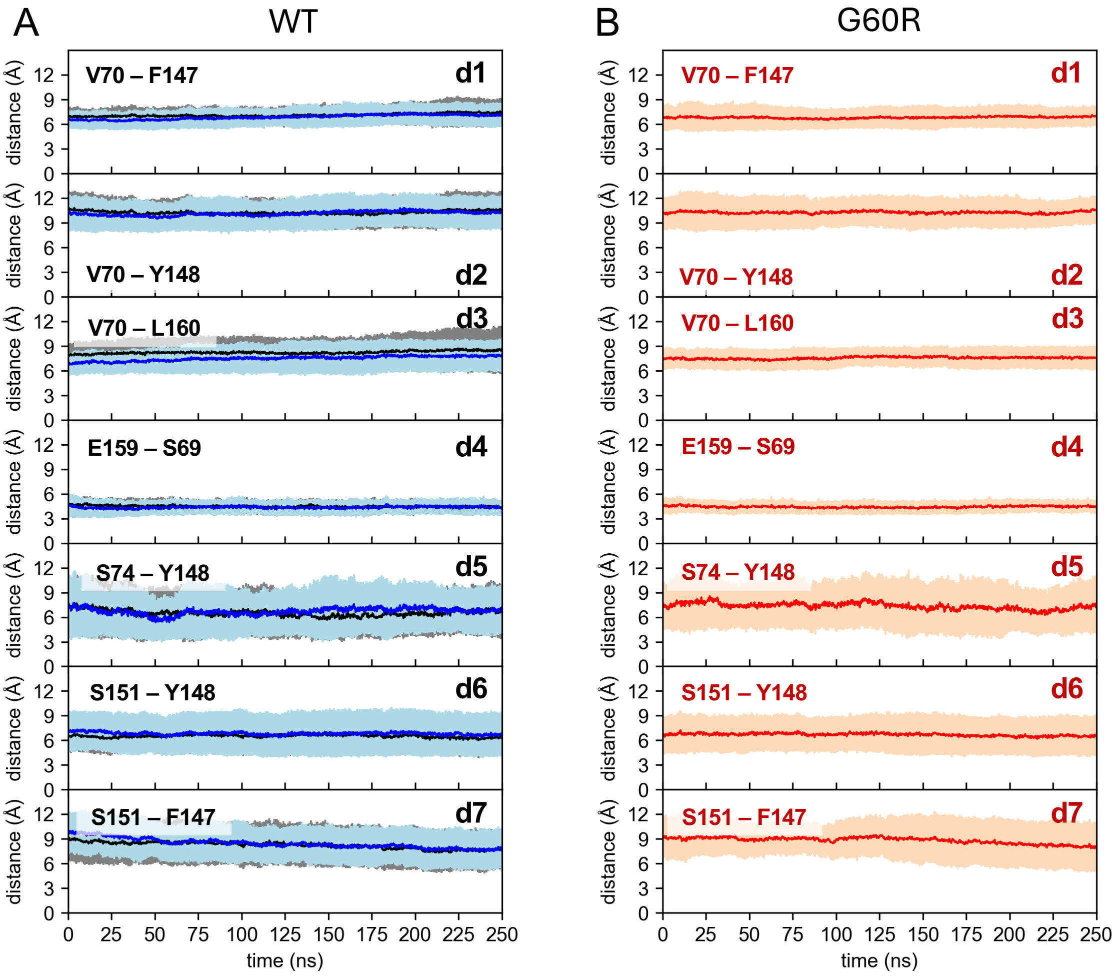

Figure S7. Inter-subunit distances in benchmark simulations. *Time evolution of distances describing the cis-linear and trans-interactions for the WT (**A, black line**) and G60R (**B, red line**) multi-Pore I. Blue lines are from the standard mass trajectories. Continuous lines and shaded areas are average values and standard deviations calculated over all realizations of the same distance in the assemblies and all benchmark trajectories.*

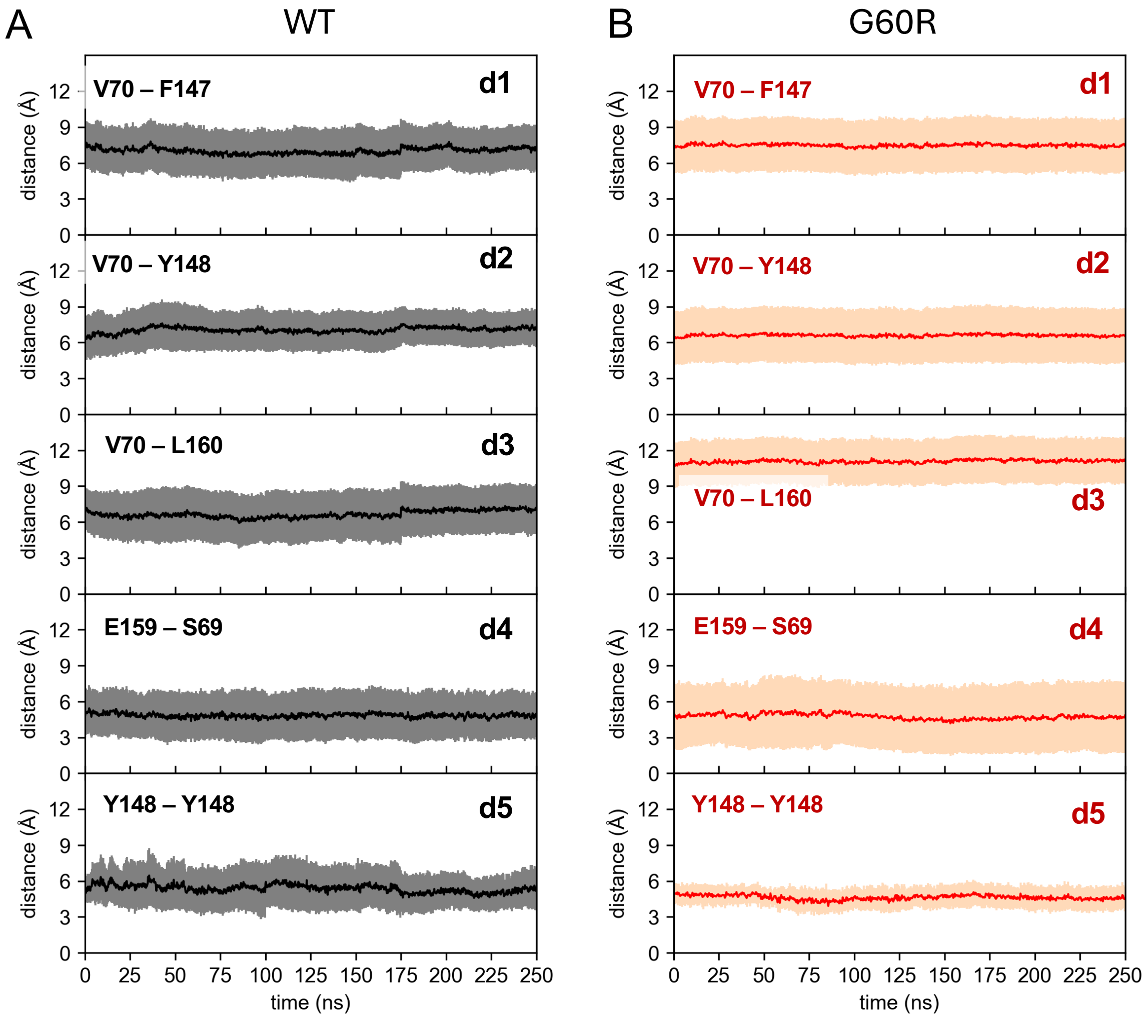

Figure S8. Inter-subunit distances in benchmark simulations. *Time evolution of distances describing the cis-linear and trans-interactions for the WT (**A**) and G60R (**B**) multi-Pore II. Continuous lines and shaded areas are average values and standard deviations calculated over all realizations of the same distance in the assemblies and all benchmark trajectories.*

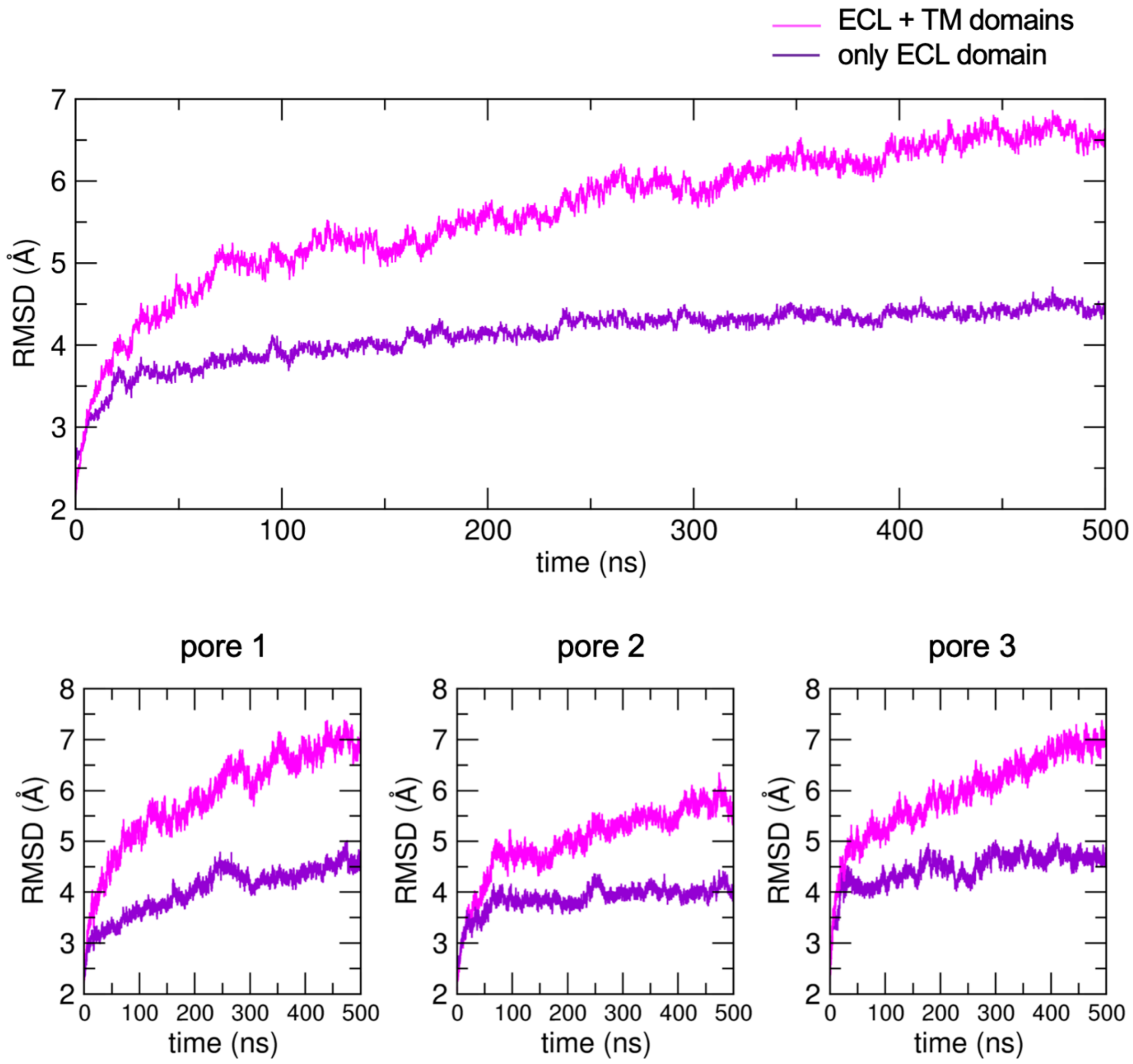

Figure S9. RMSD of the inner protomers of the multi-Pore I WT, restricted set of restraints. *The RMSD of the protein backbone is calculated for the entire system in the upper panel and for the three individual pores included in the system in the lower ones. The RMSD includes both the TM and the ECL domains (magenta profile) or considers only the ECL domain (purple profile). The four outermost protomers are kept fixed and excluded from the calculation, as described in the main text.*

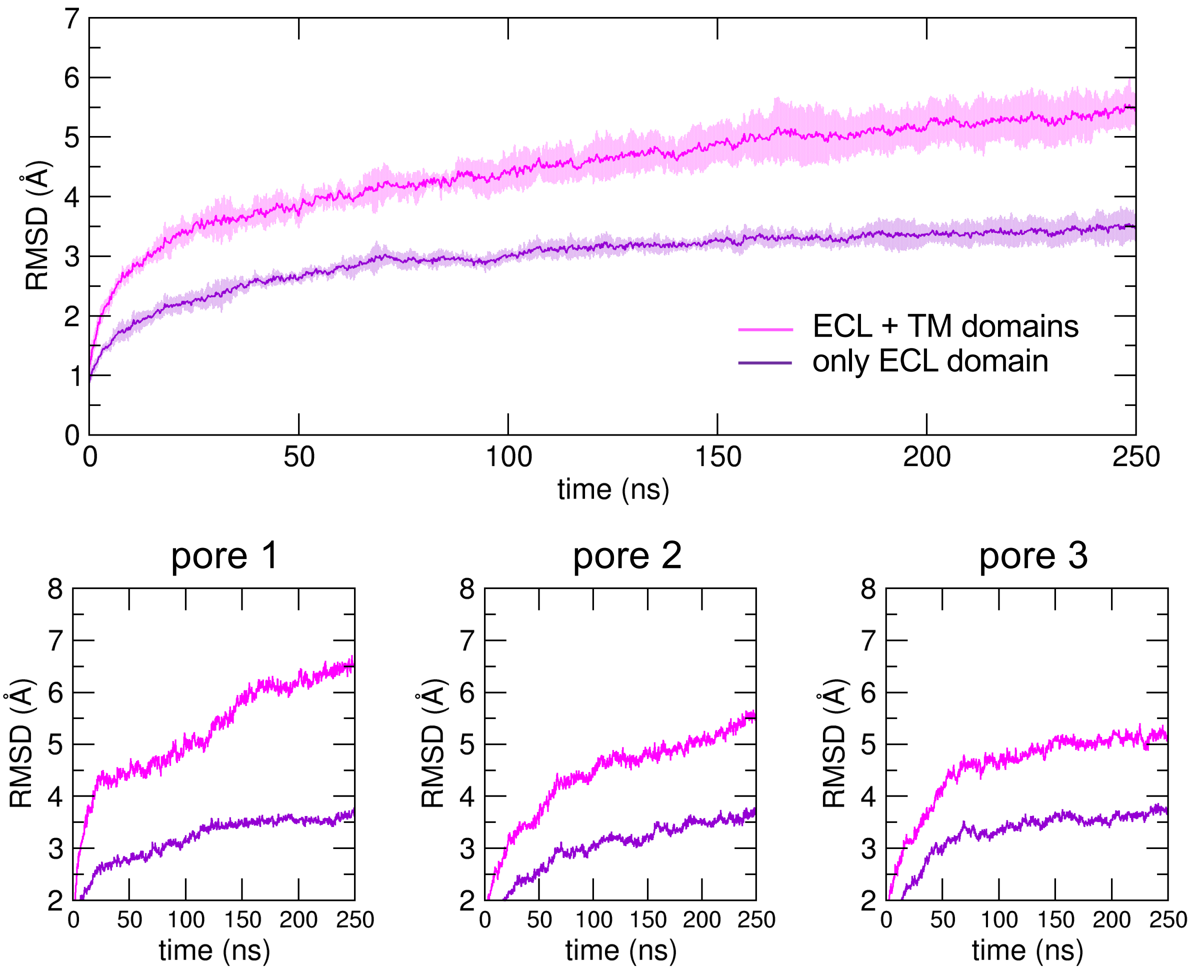

Figure S10. RMSD of the inner protomers of the multi-Pore I WT, benchmark replicas. *The RMSD of the protein backbone is calculated for the entire system in the upper panel and for the three individual pores included in the system in the lower ones. The RMSD includes both the TM and the ECL domains (magenta profile) or considers only the ECL domain (purple profile). The four outermost protomers are kept fixed and excluded from the calculation, as described in the main text.*

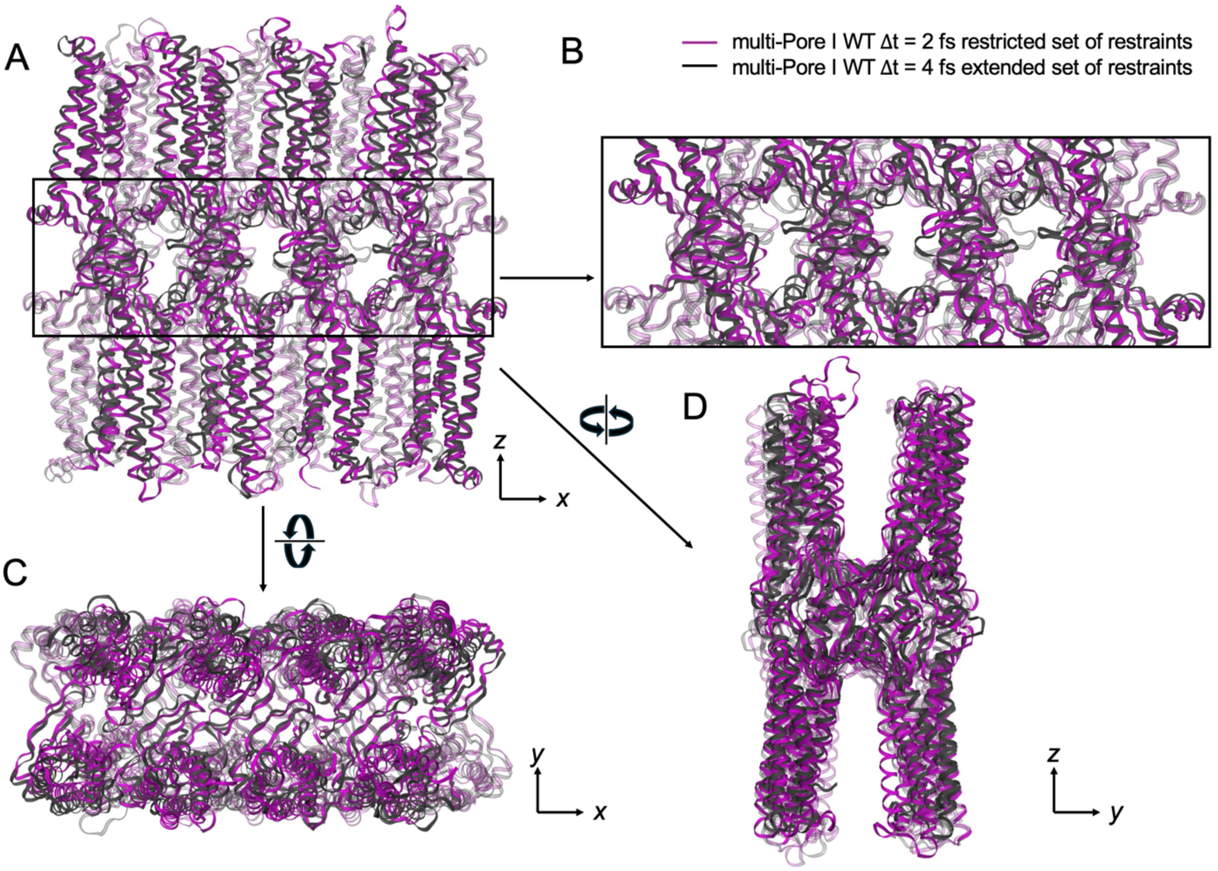

Figure S11. Superposition of the Cldn5 multi-Pore I WT configurations simulated with the extended and restricted set of restraints. *The configurations obtained after 500 ns of standard MD simulations with HMR (black) and extended set of restraints is superimposed to the configuration obtained after the same time interval in the simulations with standard masses and restricted set of restraints (purple). The systems are shown from the basolateral (**A, B**) and apical (**C, D**) views.*

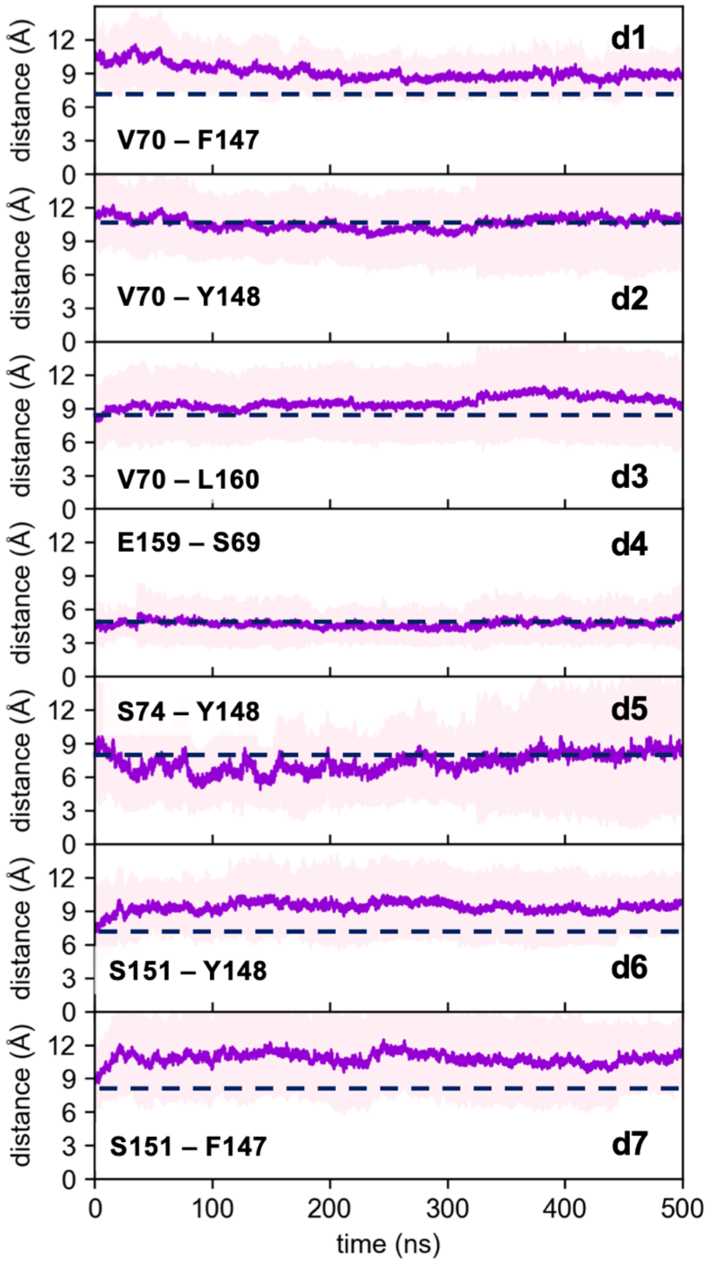

Figure S12. Cis- and trans-interactions calculated for the Cldn5 multi-Pore I WT system simulated with the restricted set of restraints. *Distances are calculated on a timescale of 500 ns of standard MD simulations and named with the same nomenclature used in the main text. The average values of distances from simulations with standard atomic masses and the extended set of restraints are reported as black dashed lines.*

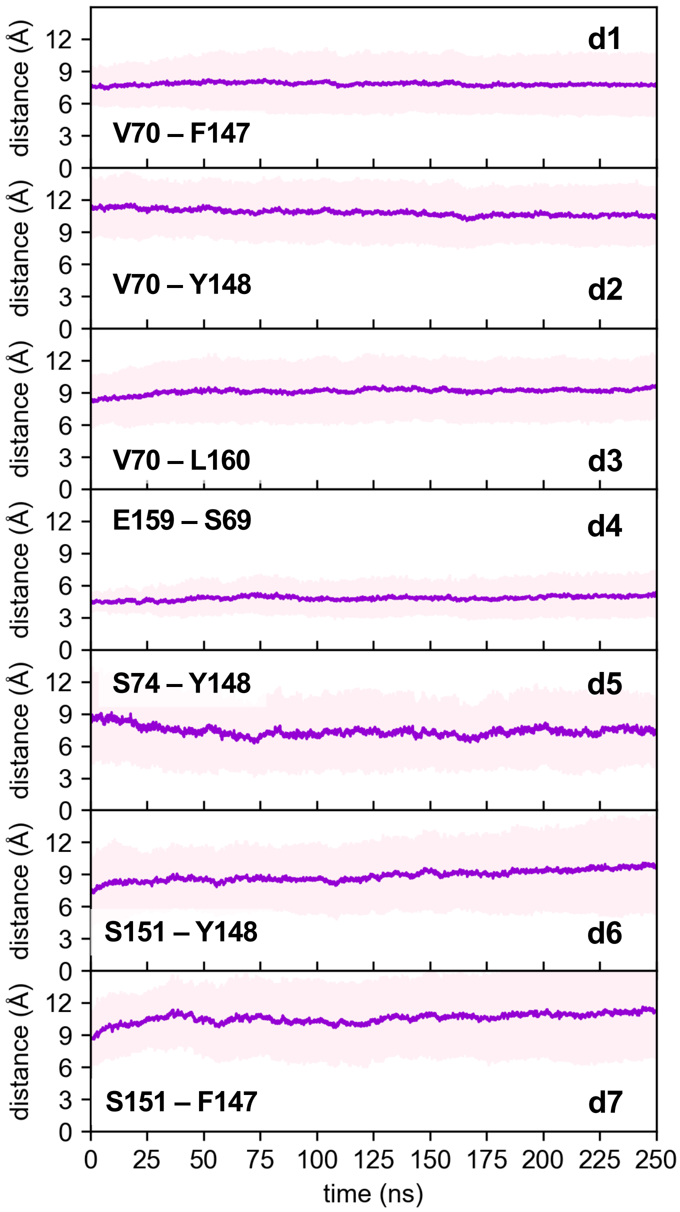

Figure S13. Cis- and trans-interactions calculated for the Cldn5 multi-Pore I WT system benchmark replicas. *The average and the standard deviation of each distance over the three replicas are reported.*

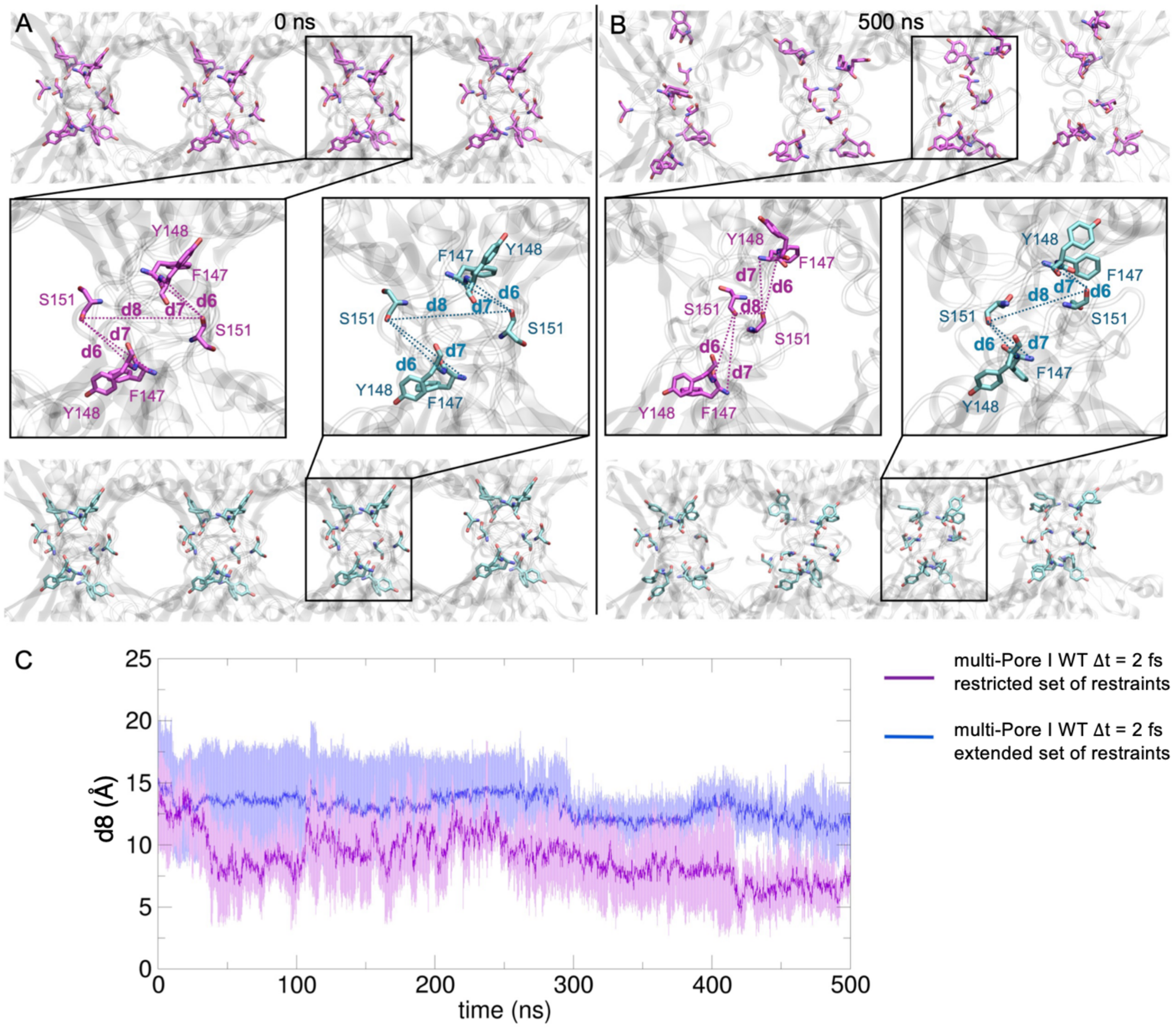

Figure S14. Modification of the trans-interactions in multi-Pore I models. *Distances between S151 and Y148 (d6), S151 and F147 (d7), and between opposing S151 residues (d8) are shown for the multi-Pore I simulated with the extended (blue structures) or restricted (purple structure) set of positional restraints and standard atomic masses. The configurations at the beginning and the end of the MD simulations are shown in panels* ***A*** *and* ***B****, respectively.* ***C****, evolution of the distance between the hydroxyl O-atoms belonging to the sidechains of opposing S151 residues (d8) in the restrained (blue profile) and unrestrained (purple profile) MD simulations. The profile and the associated error are calculated as average and standard deviation over each pair of S151 residues in the multi-Pore I models.*

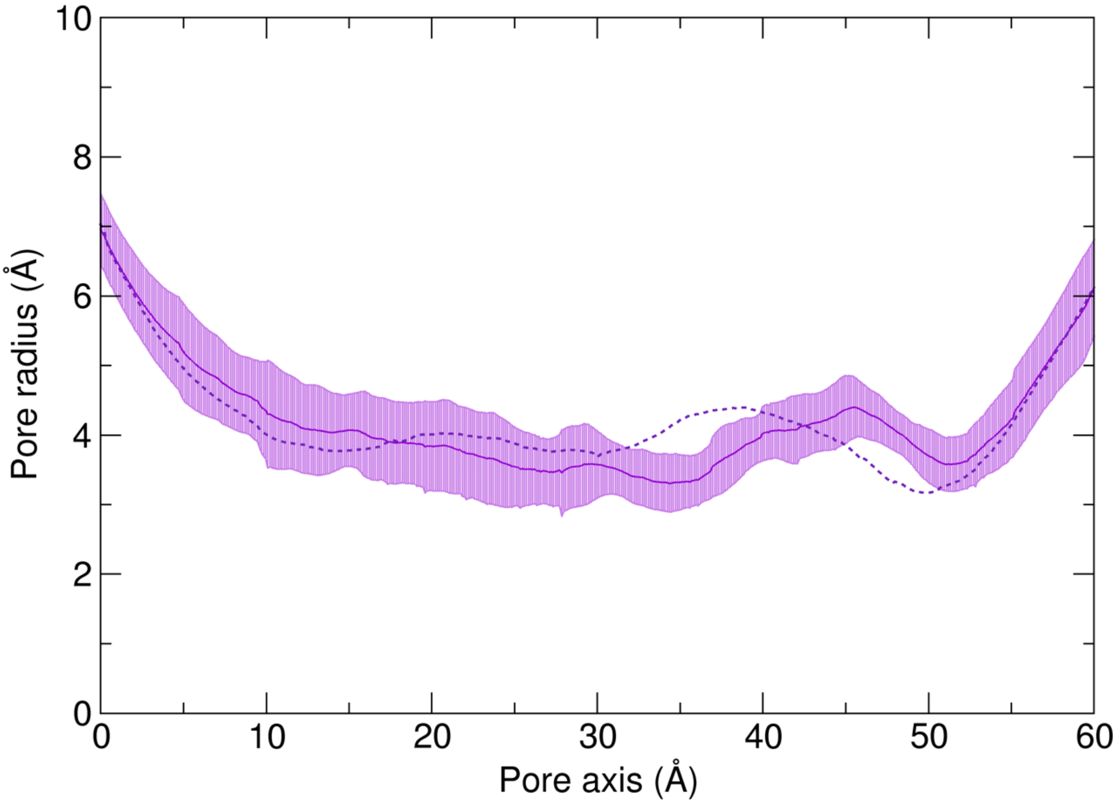

Figure S15. Pore radius profiles of the Cldn5 multi-Pore I WT system with the restricted set of restraints. *Time-average of the central pore radius (solid purple line) and associated error (shaded area) were calculated using HOLE. The dotted purple line is the average profile calculated over the three pores.*

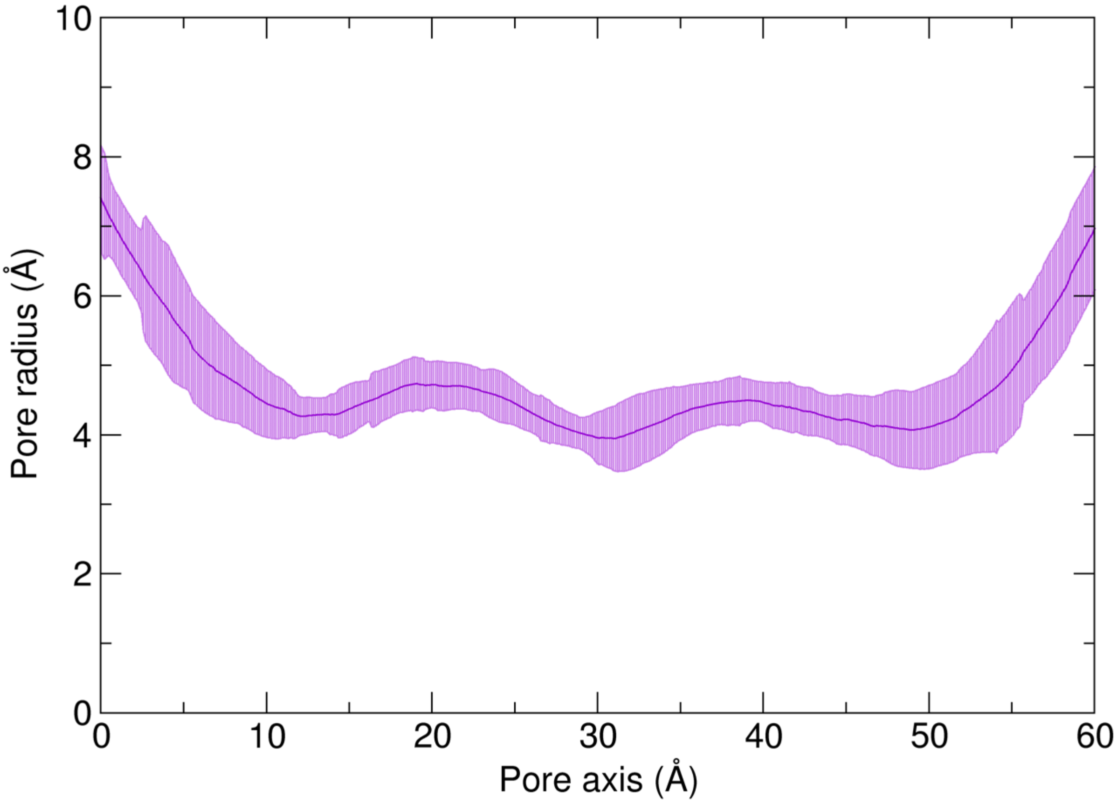

Figure S16. Pore radius profiles of the Cldn5 multi-Pore I WT system with the restricted set of restraints, benchmark replicas. *Time-average of the central pore radius (solid purple line) and associated error (shaded area) were calculated using the HOLE program. Data obtained from the combination of the three benchmark replicas.*

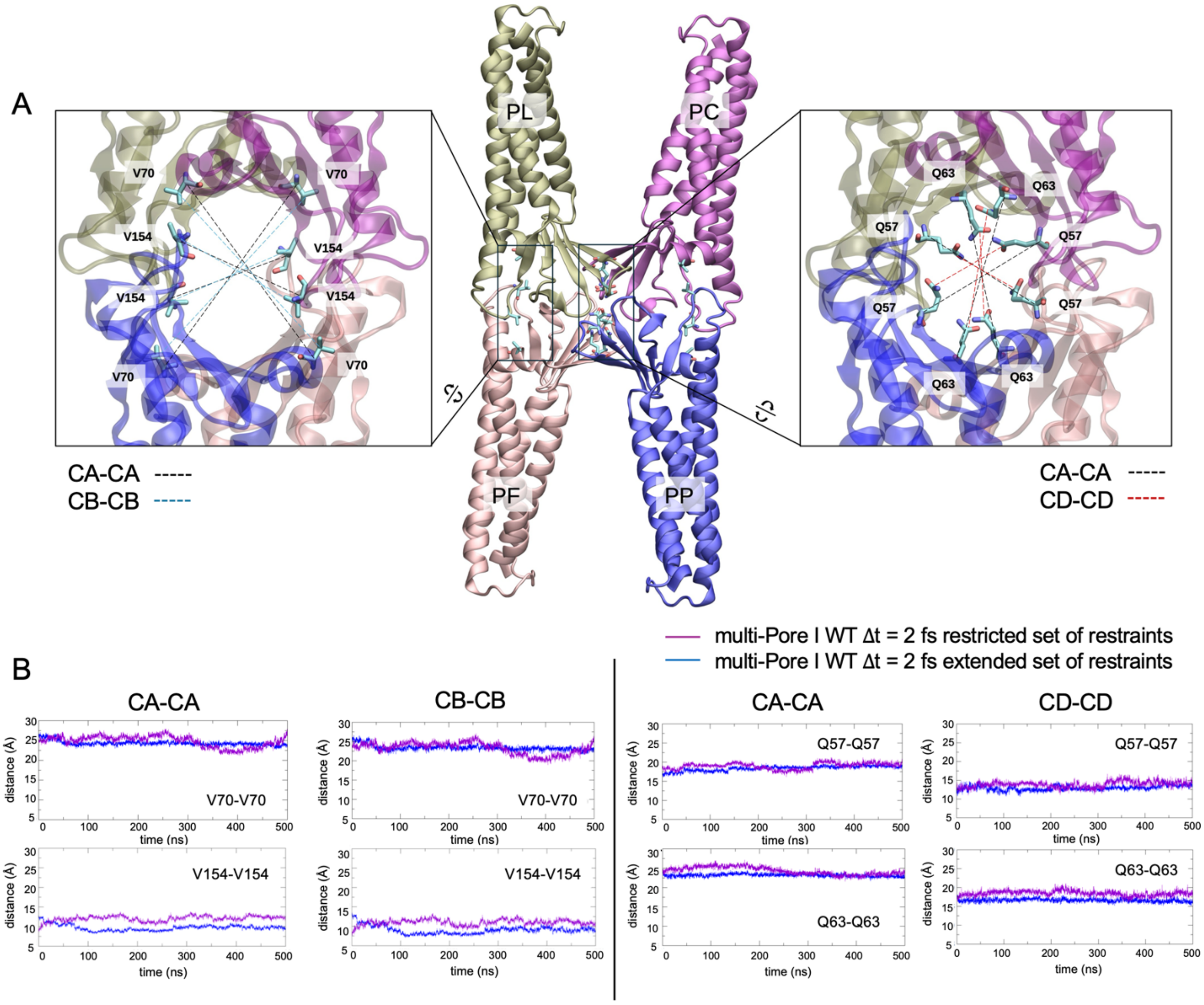

Figure S17. Stability of the multi-Pore I β-barrel during the control MD simulations with standard atomic masses. ***A***, *three-dimensional representation of the central pore in the multi-Pore I WT model. Close-up views of the two pairs of residues considered to assess the integrity of the β-barrel are shown on the left for the V154 and V70 found at the entrances of the pore, and on the right for the Q57 and Q63 pairs at the center of the cavity.* ***B***, *average distances were calculated during the MD simulations with the extended (blue) or restricted (purple) sets of restraints and a time step of 2 fs. Distances are calculated using the Cα-atoms (CA) or the most external C-atom belonging to the sidechains (CB is the Cβ , and CD is the Cδ atom of the valine and glutamine residues, respectively).*

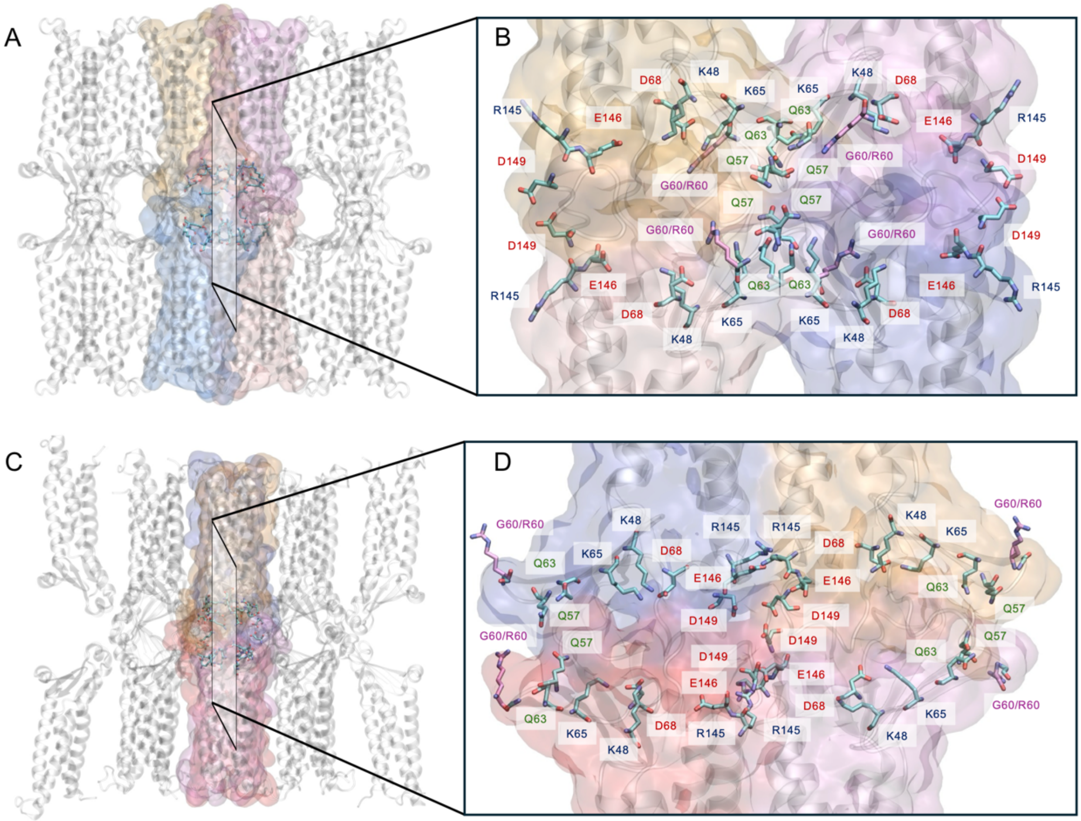

Figure S18. ***Pore-lining residues in multi-Pore I and multi-Pore II models. A****, equilibrated structure of multi-Pore I. All the Cldn5 proteins are represented using gray ribbons. Colored surfaces are added to the four Cldn5 proteins forming the central pore.* ***B****, a cross-section of the central paracellular cavity, with the side chains of the pore lining residues represented as sticks and the mutated G60R side chains colored in purple.* ***C****, equilibrated structure of multi-Pore II. All Cldn5 proteins are represented using grey ribbons. Colored surfaces are added to the four Cldn5 proteins, forming the central pore.* ***D****, cross-section of the central paracellular cavity, with the same representation of panel B.*

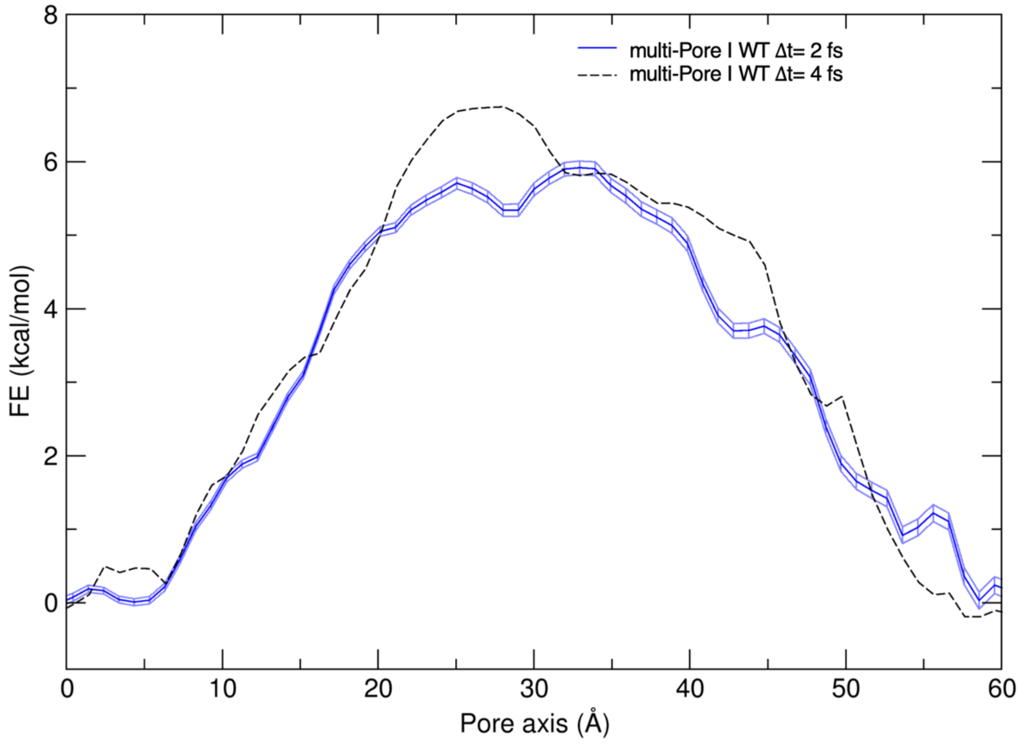

Figure S19. Free energy profile of Na^+ permeation through the Cldn5 multi-Pore I performed with a time step of 2 fs. *A representative configuration was selected after 200 ns of standard MD simulation and used to perform the FE calculation with the US-WHAM method, adopting a time step of 2 fs and using the same protocol described for the multi-Pore I WT model discussed in the main text. The FE profile obtained with HMR is also provided (black dashed line) for comparison.*

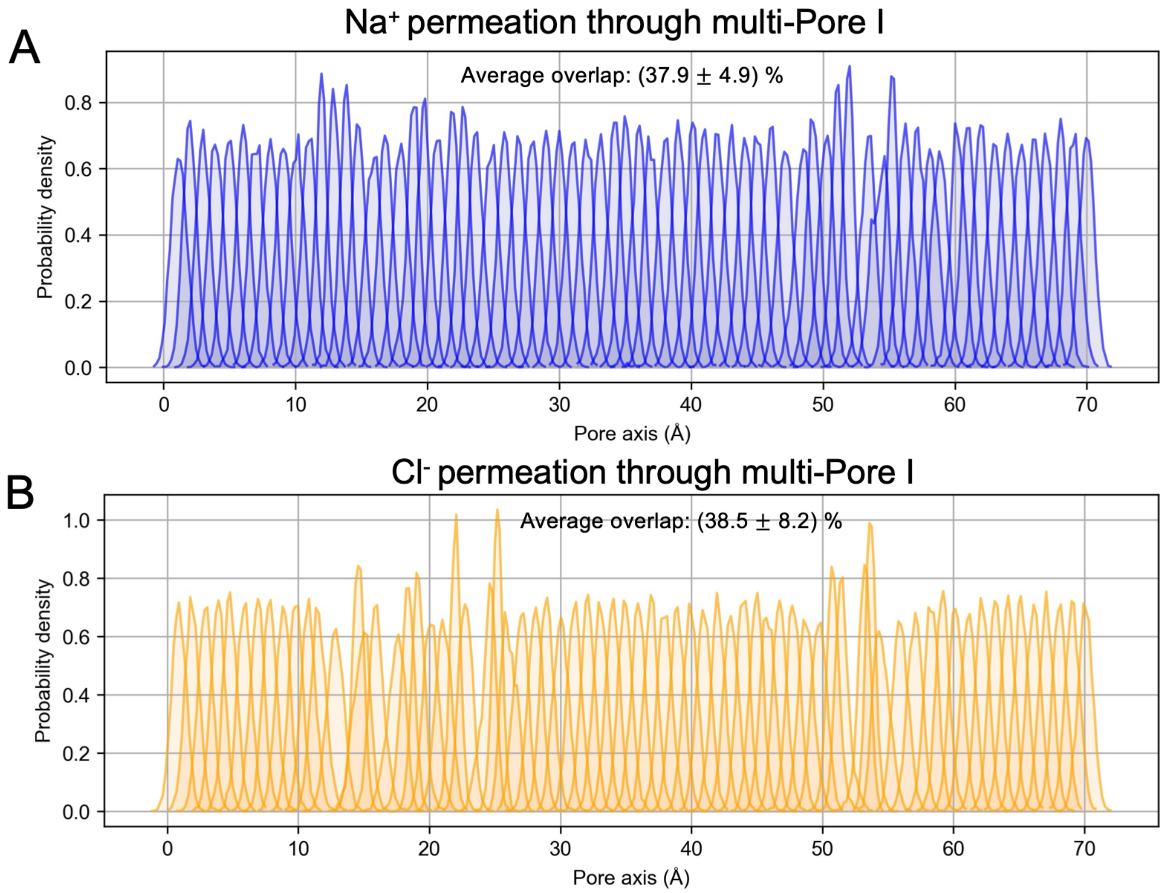

Figure S20. Overlap of the distributions of adjacent windows from umbrella sampling calculations*. Distributions of the y-coordinate (CV) in the 70 1-Å-spaced windows simulated during US calculations, for Na^+ and Cl^- permeation through the multi-Pore I central cavity are shown in panels **A** and **B**, respectively.*

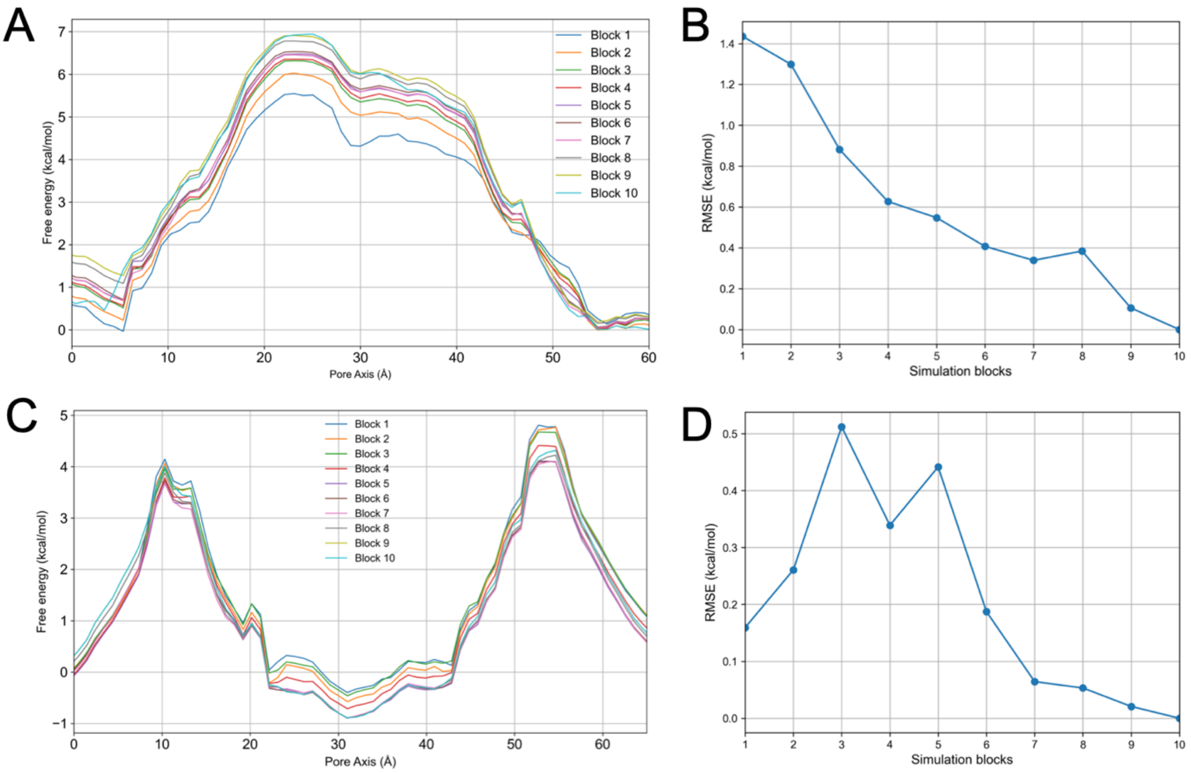

Figure S21. Convergence of the umbrella sampling calculations. *Time-cumulative block analysis and RMSE over blocks are shown in panels **A** and **B**, respectively, for* Na^+ *and in panels **C** and **D**, respectively, for* Cl^- *permeation through the multi-Pore I central cavity.*
